# PRDX2 removal inhibits the cell cycle and autophagy in colorectal cancer cells

**DOI:** 10.18632/aging.103690

**Published:** 2020-07-20

**Authors:** Xiangru Zheng, Jinlai Wei, Wenjun Li, Xiaoli Li, Wuyi Wang, Jinbao Guo, Zhongxue Fu

**Affiliations:** 1 Department of Gastrointestinal Surgery, The First Affiliated Hospital of Chongqing Medical University, Chongqing, China; 2 Department of Pharmacy, The Third Affiliated Hospital of Chongqing Medical University, Chongqing, China; 3 College of Pharmacy, Chongqing Medical University, Chongqing, China; 4 Department of Thoracic Surgery, The First Affiliated Hospital of Chongqing Medical University, Chongqing, China

**Keywords:** colorectal cancer, cell-cycle, autophagy, P38 pathway

## Abstract

Colorectal cancer (CRC) is a prevalent worldwide disease in which the antioxidant enzyme peroxiredoxin 2 (PRDX2) plays an important role. To investigate the molecular mechanism of PRDX2 in CRC, we performed bioinformatics analysis of The Cancer Genome Atlas (TCGA) datasets and Gene Expression Omnibus (GEO) DataSets (accession no. *GSE81429*). Our results suggest that PRDX2 is associated with cell-cycle progression and autophagy activated by the P38 MAPK/FOXO signaling pathway. Using a short-hairpin RNA vector, we verified that PRDX2 is essential for CRC cell proliferation and S-phase progression. Immunostaining, electron microscopy and western blotting assays verified the effect of PRDX2 knockdown on autophagy flux and p38 activation. The P38 activator dehydrocorydaline chloride partially rescued the effects of *sh-PRDX2* on the expression of proteins related to cell-cycle progression and autophagy. We verified the correlation between PRDX2 expression and the expression of an array of cell-cycle and autophagy-related genes using data from an independent set of 222 CRC patient samples. A mouse xenoplast model was consistent with in vitro results. Our results suggest that PRDX2 promotes CRC cell-cycle progression via activation of the p38 MAPK pathway.

## INTRODUCTION

In 2015 in China, there were 376,000 new cases of colorectal cancer (CRC), and the disease caused 191,000 deaths, making it one of the leading causes of cancer death for both men and women [1]. In the United States, CRC is among the 3 most prevalent cancers, with a 5-year mortality rate of 35% and a 10-year mortality rate of 42%, regardless of the administration of chemotherapy, radiation and/or surgery [2]. Unfortunately, the incidence of CRC is increasing because of poor dietary habits, smoking, low physical activity and obesity [3].

In our previous study, we determined that PRDX2 is differentially expressed in CRC tissue compared to adjacent normal tissue; increased expression is associated with poor tumor differentiation and advanced “tumor, node, metastasis” (TNM) stages [4]. We further demonstrated that PRDX2 knockdown inhibits the growth [5], stemness [6] and chemotherapy resistance [7] of CRC cells. We also revealed a regulatory loop by which PRDX2 inhibits tumor metastasis and chemotherapeutic resistance [8]. PRDX2 has also been shown to play a tumor-promoting role in CRC [9] and to have prognostic value for CRC patients [10].

High-throughput sequencing technologies, such as RNA sequencing (RNA-seq), provide efficient new methods to evaluate differential gene expression between tumors and matched nonmalignant tissues. RNA-seq is gradually altering our approach to understanding cancer. For example, application of Kyoto Encyclopedia of Genes and Genomes (KEGG) pathway analysis and drugBase exploration to RNA-seq data can reveal the function of thousands of genes involved in the development of cancers that may serve as potential therapeutic targets [11]. In the clinic, this technology has been used to diagnose and prognosticate cancers, classify tumor stages and select individualized chemotherapy drug regimens [11–13].

The mitogen activated protein kinase (MAPK) pathway is a wide-ranging impact signaling network downstream of the epidermal growth factor receptor (EGFR) that affects biological processes such as cell proliferation, cell-cycle regulation and apoptosis [14, 15]. There are three major subfamilies in the MAPK pathway: mitogen-activated protein kinase 14 (MAPK14, p38), mitogen-activated protein kinase 8 (MAPK8, JNK) and mitogen-activated protein kinase 1 (MAPK1, ERK). The activation of the MAPK pathway plays an important role in intestinal epithelial differentiation [16]. Evidence has shown that the MAPK pathway is involved in human CRC and is a potential target for treatment [17]. Furthermore, activating mutations in genes whose products are involved in the MAPK pathway are frequently detected in CRC patients [18].

To further unravel the mechanism of PRDX2 in CRC, we investigated gene expression profiles in The Cancer Genome Atlas (TCGA) through the UALCAN cancer database. Furthermore, we studied a publicly available RNA-seq data set from the Gene Expression Omnibus (GEO) database, comparing gene expression in cells transfected with *PRDX2* siRNA (*si-PRDX2*) and control cells. According to the results of this analysis, we introduced *PRDX2* shRNA (*sh-PRDX2*) and then evaluated the role and molecular mechanism of PRDX2 expression in cell-cycle regulation and autophagy both in vitro and in vivo. Finally, we characterized the relationship between P38 MAPK and PRDX2 by applying a P38 MAPK activator to rescue the PRDX2-dependent gene expression changes.

## RESULTS

### PRDX2 is highly expressed in CRC cells

**Figure 1:**
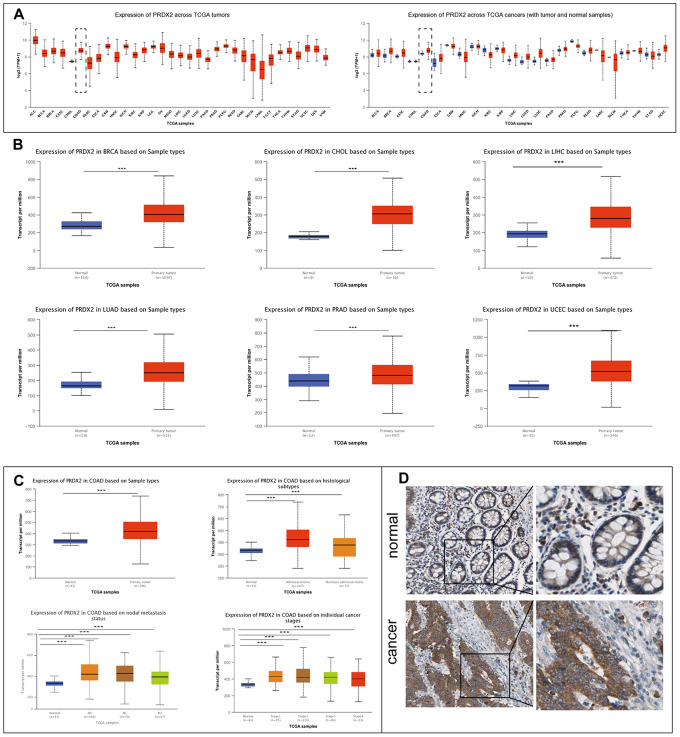
**PRDX2 is upregulated in a variety of cancers, including CRC.** (**A**) Expression of PRDX2 across TCGA tumors. Left panel: absolute expression values; right panel; expression in tumor versus normal samples. (**B**) Analysis of *PRDX2* mRNA expression levels in normal and primary tumor tissues for breast invasive carcinoma (BRCA), cholangiocarcinoma (CHOC), liver hepatocellular carcinoma (LIHC), lung adenocarcinoma (LUAD), prostate adenocarcinoma (PRAD) and uterine corpus endometrial carcinoma (UCEC). (**C**) Analysis of *PRDX2* mRNA expression levels between COAD and colon tissue according to histological subtypes, metastasis status, and individual cancer stages using the TCGA database through the UALCAN website. (**D**) Representative IHC photo images of PRDX2 protein expression in CRC tissues and normal colon tissues from The Human Protein Atlas website. ***P < 0.001 compared with the control group.

We used the UALCAN website (UALCAN: http://ualcan.path.uab.edu) to determine the expression pattern of PRDX2 in multiple tumor types compared to corresponding adjacent tissues [19]. Our analysis revealed that the mRNA expression level of PRDX2 is upregulated significantly in several cancers, including breast invasive carcinoma (BRCA), cholangiocarcinoma (CHOL), liver hepatocellular carcinoma (LIHC), lung squamous cell carcinoma (LUAD), prostate adenocarcinoma (PRAD), rectum adenocarcinoma (READ), uterine corpus endometrial carcinoma (UCEC) and colon adenocarcinoma (COAD) (Figure 1A). Comparison of the transcript-per-million values in normal and primary tumor tissues verified PRDX2 overexpression in BRCA, CHOL, LIHC, LUAD, PRAD and UCEC (Figure 1B). COAD and READ are subtypes of CRC. We further investigated the overexpression of PRDX2 in COAD and found that PRDX2 expression was high across multiple tumor histological subtypes, cancer nodal metastasis status and individual cancer stages (Figure 1C). The expression of PRDX2 in READ was consistent with that in COAD (Supplementary Figure 1). Moreover, PRDX2 protein levels are upregulated in CRC tissues according to The Human Protein Atlas (https://www.proteinatlas.org) (Figure 1D) [20]. These results suggest that PRDX2 is upregulated during carcinogenesis and may increase the malignancy of CRC.

### PRDX2 depletion is associated with FOXO pathway enrichment

**Figure 2:**
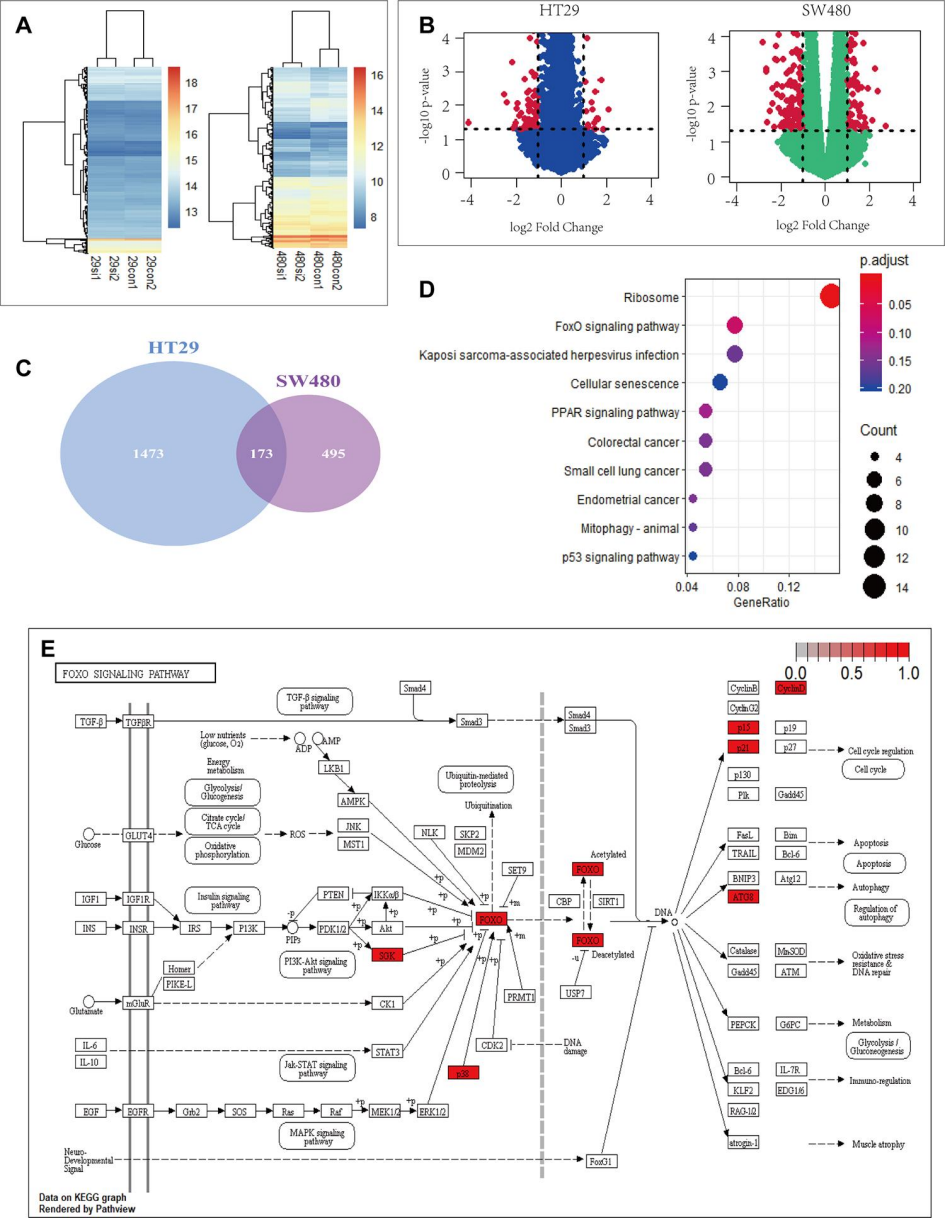
**PRDX2 is associated with cell-cycle and autophagy regulation through the P38/FOXO pathway.** (**A**, **B**) Heat maps (**A**) and volcano plots (**B**) showing differential gene expression upon PRDX2 depletion in HT29 and SW480 CRC cells; data from the GSE81429 dataset. (**C**) Venn diagram showing that 173 gene are differentially expressed in both HT29 and SW480 cells. (**D**) KEGG pathway enrichment of differently expressed genes in HT29 and SW480 cells. (**E**) FOXO pathway with differentially expressed genes in HT29 and SW480 cells.

To gain mechanistic insight into the role of PRDX2 in CRC, we used RNA-seq data from GEO DataSets (accession no. *GSE81429*) for HT29 and SW480 cells depleted of PRDX2 by *si-PRDX2*. Gene expression profile analysis using heatmaps (Figure 2A) and volcano plots (Figure 2B) showed 1646 and 668 differentially expressed genes from HT29 and SW480 cell lines, respectively. One hundred and seventy-three genes were significantly differentially expressed in both cell lines (Figure 2C and Supplementary File 1). To further evaluate the functions of the 173 genes, we performed KEGG enrichment analysis using RStudio (Figure 2D) [21]. Among the top 10 differentially expressed pathways, the FOXO pathway was the second most significantly enriched (Figure 2E). Notably, *p38 MAPK* was identified within this pathway as a significantly differentially expressed gene. These results raise the possibility that PRDX2 may regulate cell-cycle progression and autophagy in CRC through the p38/FOXO pathway.

### Knockdown of PRDX2 suppresses CRC cell-cycle progression

**Figure 3:**
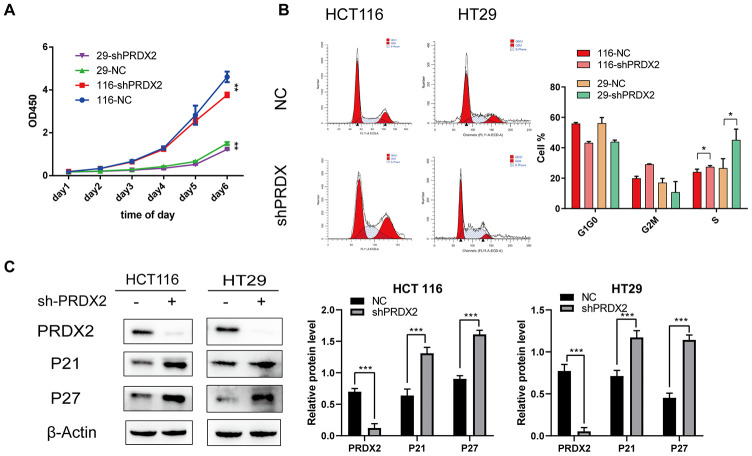
**Effects of PRDX2 knockdown on the cell cycle.** (**A**) Cell proliferation was markedly inhibited after PRDX2 downregulation as determined by CCK-8 assay. (**B**) Flow cytometry cell-cycle analysis showed that sh-PRDX2 induces cell-cycle arrest in HT29 and HCT116 cells. (**C**) Representative western blot images of the effect of sh-PRDX2 on the expression levels of PRDX2, P21, and P27. β-actin was used as a loading control. The data are shown as the mean ± SD of three experiments. *P < 0.05, **P < 0.01, ***P < 0.001 compared with the control group.

To explore whether PRDX2 expression regulates the cell cycle in CRC cells, we introduced PRDX2-shRNA into HCT116 and HT29 cell lines and evaluated proliferation using a cell-counting kit (CCK-8) assay. The data suggest that PRDX2 knockdown suppresses CRC cell proliferation (Figure 3A). Furthermore, flow cytometry results indicate that PRDX2 knockdown causes cell-cycle arrest in S phase (Figure 3B). Western blot analysis demonstrated that *sh-PRDX2* upregulates the expression of the cell-cycle markers P21 and P27 in both HCT116 and HT29 cell lines (Figure 3C and Supplementary Figure 3). However, high expression of PRDX2 did not significantly affect the cell cycle (Supplementary Figure 2A, 2C). These results indicated that PRDX2 plays a critical role in maintaining CRC cell-cycle progression.

### Loss of PRDX2 function impairs autophagy flux

**Figure 4:**
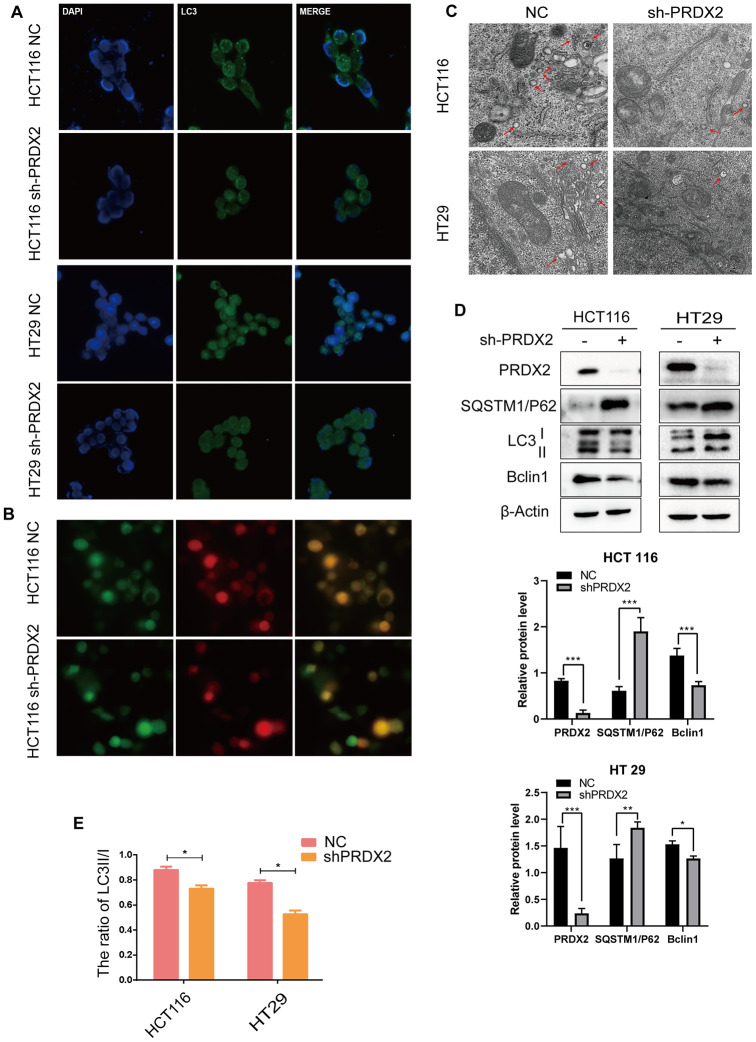
**Knockdown of PRDX2 suppresses CRC cell autophagy flux.** (**A**) Immunofluorescence images of GFP-labeled LC3B staining in *sh-PRDX2* and control CRC cells. (**B**) NC and *sh-PRDX2* cells were transfected with GFP-RFP-LC3 plasmids. (**C**) *Sh-PRDX2* and control CRC cells were analyzed by transmission electron microscopy. Arrows indicate autophagosomes. (**D**) Western blot of autophagy-related proteins (LC3B, SQSTM1/P62, Beclin 1) in *sh-PRDX2* and control CRC cells. (**E**) The ratio of LC3 II/I. The data are shown as the mean ± SD of three experiments. *P < 0.05, **P < 0.01, ***P < 0.001 compared with the control group.

Based on the above GEO data, we also evaluated the effect of *sh-PRDX2* on autophagy in HCT116 and HT29 cell lines. Cells were stained for LC3B expression with GFP and infected by cherry-GPF-LC3 plasmid. Immunofluorescent images revealed a decrease in GFP dots in sh-PRDX2 cells, suggesting that PRDX2 knockdown impaired autophagic flux (Figure 4A, 4B). Electron microscopy of the ultrastructural morphology verified that the number of autophagosomes in PRDX2-shRNA cells was decreased (Figure 4C). We further performed western blotting to measure the protein expression patterns of LC3, Beclin 1 and SQSTM1/P62. SQSTM1/p62 was increased in PRDX2 knockdown cells, while the ratio of LC3B II/I (Figure 4E and Supplementary Figure 4) and Beclin 1 were decreased, further confirming that PRDX2 knockdown blocks autophagosome formation (Figure 4D). Furthermore, overexpression of PRDX2 promoted autophagy flux (Supplementary Figure 2B, 2C). Our observations demonstrated that the balance of autophagy flux is impaired in *sh-PRDX2* cells, confirming that PRDX2 is involved in autophagy flux in CRC cells.

### PRDX2 mediates its effect through the P38 MAPK Pathway

**Figure 5:**
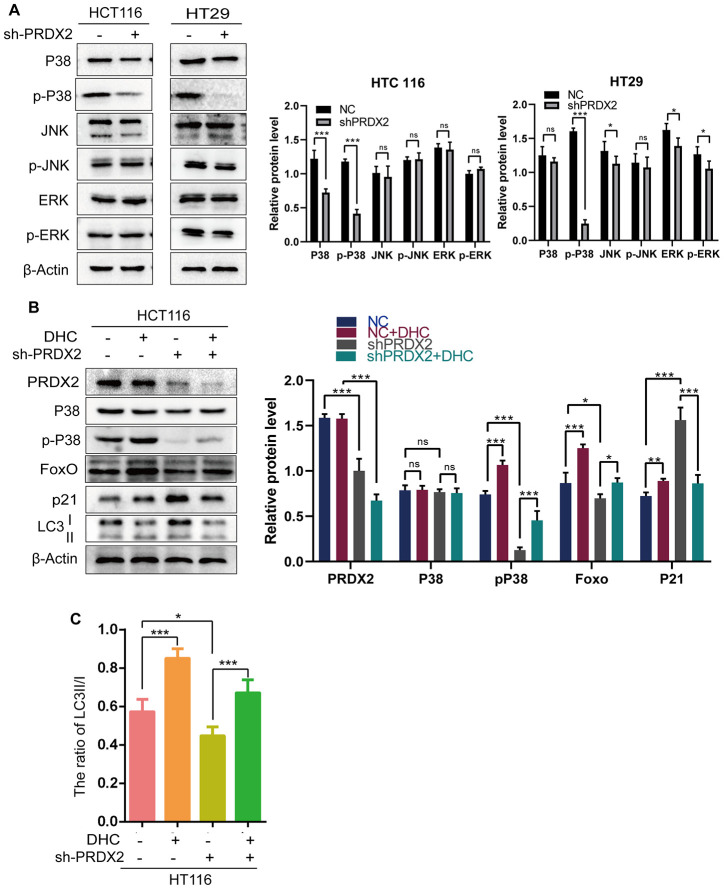
**The effects of PRDX2 on the cell cycle and autophagy are mediated by the P38 pathway.** (**A**) Western blots of MAPK signaling-related proteins. (**B**) Western blots of proteins related to the P38 pathway (P38 and p-P38), cell-cycle regulation (P21) and autophagy (LC3B) in *sh-PRDX2* and control CRC cells with or without exposure to DHC (1 μM) for 24 h. (**C**) The ratio of LC3 II/I. The data are shown as the mean ± SD of three experiments. *P < 0.05, **P < 0.01, ***P < 0.001 compared with the control group.

Evidence suggests that the P38 MAPK pathway downregulates autophagy [22], and our RNA-seq data suggests that P38 is differentially expressed upon PRDX2 depletion. Therefore, we analyzed the expression of MAPK signaling-related proteins in *sh-PRDX2* cells. The protein levels of phospho-P38 MAPK were significantly decreased in PRDX2-knockdown cells, though levels of p-JNK and p-ERK did not appear to be significantly affected (Figure 5A and Supplementary Figure 5). Moreover, 24-hour treatment of CRC cells with 1 μM of the p38 MAPK activator dehydrocorydaline chloride (DHC) elevated phospho-p38 MAPK and suppressed the levels of p21 and the ratio of LC3B II/I expression in PRDX2 knockdown cells (Figure 5B, 5C and Supplementary Figure 6). These results indicate that PRDX2 acts via the P38/FOXO pathway to regulate cell-cycle progression and autophagy flux.

### PRDX2 expression is correlated with the expression of cell-cycle and autophagy genes in CRC patients

**Figure 6:**
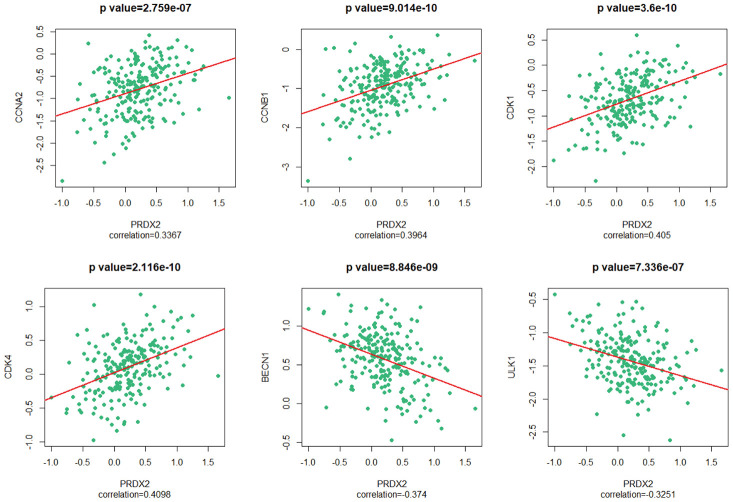
**PRDX2 is associated with the cell-cycle and autophagy phenotypes of CRC.** Scatter plot showing the correlation between the mRNA level of *PRDX2* and those of *CCNA2, CCNB1, CDK1, CDK4, BECN1* and *ULK1*.

To confirm our results, we investigated links between PRDX2 and cell-cycle and autophagy genes in CRC patients using the CGDSR-R and mRNA (microarray) data from an independent TCGA colorectal adenocarcinoma cohort consisting of 222 samples [23]. Our analysis revealed that the mRNA level of *PRDX2* correlated with the mRNA levels of *CCNA2* (*r* = 0.3367, *P* = 2.759e-07), *CCNB1*(*r* = 0.3964, *P* = 9.014e-10), *CDK1* (*r* = 0.405, *P* = 3.6e-10), *CDK4* (*r* = 0.4098, *P* = 2.116e-10), *BECN1* (*r* = −0.374, *P* = 8.8846e-09) and *ULK1* (*r* = −0.3251, *P* = 7.336e-07) (Figure 6). These data verify the association between PRDX2 and markers of the cell-cycle and autophagy in CRC patients.

### PRDX2 knockout inhibits CRC cell-cycle progression and autophagy flux in mice

**Figure 7:**
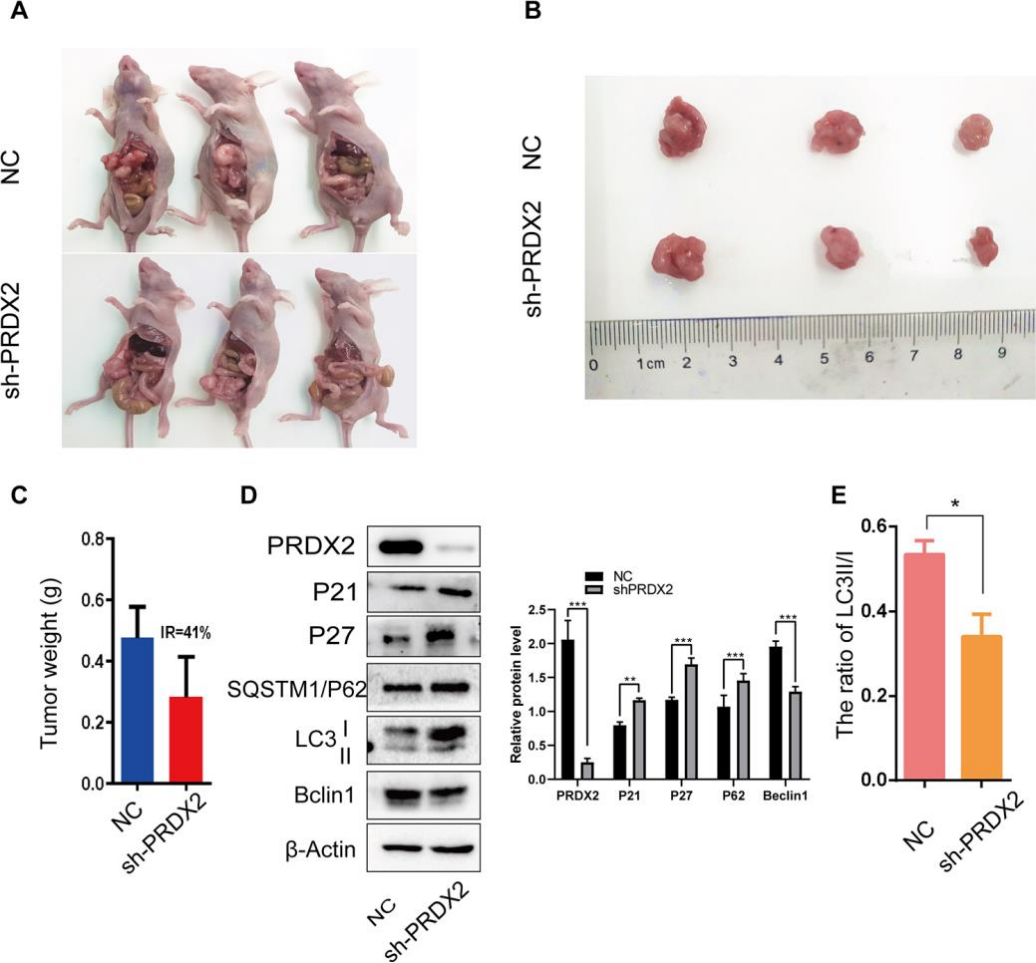
***Sh-PRDX2* inhibits cell-cycle arrest and autophagy flux in vivo.** (**A**) NC and *sh-PRDX2* cells were transplanted in the mesenteric artery at the distal end of cecum of mice for 4 weeks. (**B**) The tumors were excised from the mesenteric artery. (**C**) Tumor weights were measured. IR: the tumor growth inhibition rate = (the average tumor weight of NC group - the average tumor weight of *sh-PRDX2* group) / the average tumor weight of NC group. (**D**) Western blots of cell-cycle (left) and autophagy-related (right) proteins expressed in tumor cells. (**E**) The ratio of LC3 II/I. The data are shown as the mean ± SD . *P < 0.05, **P < 0.01, ***P < 0.001 compared with the control group.

To determine whether PRDX2 also acts as an inhibitor of the cell-cycle and autophagy in vivo, a xenoplastic model was established in nude mice. In total, 5 mice were assigned to each group; however, 2 mice in each group died within 3 days of tumor transplant. All mice died of surgery-related diseases rather than tumors. Therefore, we evaluated transplants from the 3 pairs of surviving mice. *Sh-PRDX2* tumors were significantly smaller than control tumors (Figure 7A–7C). Furthermore, western blot assays showed that downregulation of PRDX2 promoted the protein levels of P21, P27, SQSTM1/P62, as well as the ratio of LC3B II/I, and inhibited protein levels of Beclin 1 (Figure 7D, 7E). These results were consistent with our in vitro assay results and demonstrate that PRDX2 depletion inhibits the cell cycle and autophagy flux of CRC cells both in vitro and in vivo.

## DISCUSSION

Our previous studies introduced the role of PRDX2 in CRC. In this study, we further investigated the role of PRDX2 in CRC and its functional consequences. We found the following: (i) PRDX2 is highly expressed in CRC and other cancers, (ii) depletion of PRDX2 mediates p38/FOXO-pathway regulation of the cell cycle and autophagy, (iii) loss of PRDX2 causes cell arrest in S phase and impairment of autophagy flux, (iv) PRDX2 is associated with cell-cycle and autophagy phenotypes in CRC patients, and (v) PRDX2 functions through the p38/FOXO pathway to impact the cell cycle and autophagy in an in vivo CRC mouse model.

PRDX2 is a multi-functional gene in colorectal cancer and has potential to be a therapeutic target in CRC treatment. PRDX2 has been shown to play a tumor-promoting role in CRC [9] and to have prognostic value for CRC patients [10]. In our previous studies, the loss of PRDX2 inhibited the growth [5], stemness [6] and chemotherapy resistance [7] of CRC cells. We also revealed a regulatory loop by which the loss of PRDX2 inhibits tumor metastasis and chemotherapeutic resistance [8]. In the present study, our results suggest that PRDX2 loss inhibits the cell cycle and autophagy. Deleting PRDX2 or blocking the function of PRDX2 in colorectal cancer may yield efficacious results.

In the present study, we first investigated the function of PRDX2 in CRC using a public database. Our results showed that PRDX2 is not only highly expressed in CRC but also in other cancers, suggesting that our studies in colorectal cancer could provide a reference for other cancers. We also found that under all pathological conditions, PRDX2 is highly expressed in cancerous tissues compared with normal tissues. PRDX2 could therefore be easily recognized by a targeted drug, thereby protecting normal tissues.

PRDX2 may play a different role in the cell cycle of CRC tissues versus normal tissues. In bone marrow cells, a small-interfering RNA targeting PRDX2 caused G1-phase arrest in cells isolated from BALB/c mice [24]. Additionally, depletion of PRDX2 has been shown to impair G1-phase progression and to inhibit cell proliferation in human keratinocytes cells [25]. The effect of PRDX2 on the cell cycle in CRC has not been investigated until now. Our present study showed that PRDX2 depletion induced S-phase arrest rather than G1-phase arrest. TCGA database analysis positively identified PRDX2 as a cell-cycle molecule. S-phase arrest typically corresponds to DNA damage and leads cells to become more susceptible to the cytotoxic effects of radiotherapy, so therapies targeting PRDX2 may make radiation therapy more effective [26, 27].

Apoptosis is usually linked to autophagy, the other major form of cell death [32]. Autophagy is a highly conserved cellular catabolism process that balances the cellular energy by decomposing intracellular components that may cause damage [28]. Related studies have found that autophagy is a double-edged sword in the occurrence and development of tumors. On the one hand, autophagy can inhibit the canceration of cells by decomposing toxic substances that reduce gene stability and the normal replication of cells. On the other hand, autophagy reduces the abnormally elevated reactive oxygen species (ROS) levels in tumor cells, decreases the apoptosis caused by oxidative stress and promotes the growth of tumors [29–31].

In our previous study, downregulated PRDX2 induced the apoptosis of CRC cells [5]. In the present study, cells without PRDX2 showed decreased ratios of LC3II/I, increased expression of P62 and simultaneously, decreased expression of Beclin 1. These findings suggest that removal of PRDX2 may disrupt the protective effect of autophagy on CRC cells, thereby increasing apoptosis in CRC cells. Similar results have been observed in PRDX2^-/-^ mice [33]. The mechanism behind these results may involve the induction of ROS followed by the activation of mTOR, which subsequently inhibits autophagy [34].

P38 is an important regulatory pathway in tumor cells. P38 MAPK regulates cell-cycle arrest in S phase, as well as autophagy flux [29, 35, 36]. It also inhibits basal and starvation-induced autophagosome formation [37]. Data also suggest that the P38 pathway may induce autophagosome formation and increase autophagic flux [29], which is consistent with our findings that phospho-P38 is reduced upon deletion of PRDX2, inhibiting autophagy flux.

In conclusion, this study showed that in CRC cells, loss of PRDX2 inhibited cell proliferation and arrested cells in S phase. Removal of PRDX2 also inhibited autophagy flux through the p38 MAPK pathway in HT29 and HCT116 cells, suggesting that PRDX2 could be a multifunction molecule and a promising therapeutic target for CRC treatment.

## MATERIALS AND METHODS

### Bioinformatic analysis

RNA-seq data were downloaded from GEO database (accession no. *GSE81429*). After transformation with an SRA Toolkit and standard quality checking with FastQC, the RNA-seq data were aligned to the human genome (hg19 from UCSC) using HISAT2. Read count tables were generated using HTSeq-count. All software was run on Bioconda from a cloud computer (Tencent Company, China), which dramatically enhanced the analytic capabilities. Subsequent steps were conducted in RStudio (Version 1.2.1335) on our own computer. Differentially expressed genes were identified using DESeq2 with the adjusted *P* value < 0.01. Further analyses were performed using the clusterProfiler package (Version 3.4.4) and KEGG pathways (Version 3.2.3).

### CRC cell lines and cell culture

The human CRC cell lines HCT116, HT29 and human embryonic kidney 293T (293T) cells were purchased from Shanghai Zhong Qiao Xin Zhou Biotechnology Co., Ltd. The HCT116 and HT29 cell lines were cultured in McCoy's 5A Medium (Biological Industries), and the 293T in EMDM Medium supplemented with 10% fetal bovine serum (Biological Industries) at 37°C in 5% CO_2_.

### Lentiviral constructs for transfection of CRC cells

Human shRNA constructs for lentivirus packaging were purchased from Clontech (Mountain View, CA). The following target sequence was used to silence PRDX2 expression in human cells: 5'-GTGAAGCTGTCGGACTACAAA-3'. The negative control (NC) sequence was the following: 5'-GTGACGCTGTCGGACGACAAA-3.

CRC cells were plated into 24-well plates at a density of 1.5 × 10^4^ cells per well on the day before transfection with lentiviral constructs. TransIntroTM PL Transfection Reagent (TransGen Biotech, Beijing, China) at a concentration of 5 μL/mL was used to enhance the efficiency of transfection. Infected cells were maintained in McCoy's 5A medium for 12 hours, and the medium was replaced with fresh McCoy's 5A medium supplemented with 10% FBS. Puromycin was used to select the transfected cell at a concentration of 2 ug/mL for HTC116 cells and 8 ug/mL for HT29 cells.

### Antibodies and reagents

Antibodies included anti-PRDX2 (Proteintech, 10545-2-AP), anti-P21 (Cell Signaling Technology, 2947), anti-P27 (Bimake, A5053), anti-LC3 (Abcam, ab192890), anti-P6/2SQSTM1 (Proteintech, 18420-1-AP), anti-Beclin 1 (Proteintech, 11306-1-AP), anti-FOXO3A (Cell Signaling Technology, 12829), anti-P38 MAPK (Cell Signaling Technology, 8690), anti-phospho-P38 MAPK (Cell Signaling Technology, 4511), anti-JNK (Cell Signaling Technology, 9252), anti-phospho-JNK (Cell Signaling Technology, 9255), anti-ERK (Cell Signaling Technology, 4695), anti-phospho-ERK (Cell Signaling Technology, 4370) and HRP-conjugated second antibody (Proteintech, SA00001-2). (Supplementary Table 1) P38 MAPK activator (HY-N0674A) was purchased from MedChemExpress (Monmouth Junction, NJ).

### CCK-8 assay

The cell-counting kit (CCK-8) was purchased from Bimake.com (B34302). Cells were plated into 96-well plates at a density of 500 cells per well in 200 μl complete medium. Before measurement, the medium was changed to serum-free medium mixed with CCK-8 at a ratio of 9:1 and incubated for 2 hours at 37°C. The optical density values were measured on a microplate reader (Thermo, Varioakan LUX) at 450 nm on days 1, 2, 3, 4, 5, and 6.

### Protein extraction and western blotting

Cells were lysed on ice for 30 min and vortexed for 10 min in RIPA buffer (Beyotime Biotechnology, P0013B) supplemented with a phosphatase and protease inhibitor cocktail (Bimake, B15001 and B14011). The protein concentrations were measured using bicinchoninic acid (BCA) assays according to the manufacturer’s instructions (Beyotime Biotechnology, P0012). Lysed proteins were heated in a 95°C bath for 5 minutes after mixing with loading buffer (Beyotime Biotechnology, P0015). The proteins were then separated by SDS-PAGE and transferred onto PVDF membranes. The membranes were incubated in QuickBlock Blocking Buffer for Western Blots (Beyotime Biotechnology, P0252) for 15 minutes and then incubated with primary antibody dissolved in QuickBlock Primary Antibody Dilution Buffer for Western Blots (Beyotime Biotechnology, P0256; dilution ratio, 1:1,000) for 2 hours at room temperature. After three, 5-minute washes in TBS-Tween 20, the membranes were incubated with HRP-conjugated secondary antibody (dilution ratio, 1:10,000) for 2 hours at room temperature. Finally, the membranes were washed and developed with an enhanced chemiluminescence (ECL) detection reagent (Beyotime Biotechnology, P0018S). Signals were detected using the Bio-Rad ChemiDoc™ XRS+ System.

### Flow cytometry of the cell cycle

Cells were harvested and prepared according to the manufacturer’s instructions (Beyotime Biotechnology, C1052). Briefly, the cells were fixed in 70% cold ethanol for 24 hours and stained using propidium iodide at 37°C for 30 minutes in darkness. The stained cells were detected with the CytoFLEX System (Beckman Coulter) and analyzed using ModFit (Version LT V5.0.2).

### Immunofluorescence

Cells were plated on 24-well plates at a concentration of 3 × 10^4^ per well before experimentation. The medium was removed, and the cells were fixed in 500 μL of 100% methanol (precooled to −20°C overnight) for 15 minutes. The cells were then washed with PBS 3 times (5 minutes each) and incubated in PBS with 5% normal goat serum (Beyotime, China, C0265) and 0.3% Triton X-100 (Beyotime, China, ST795). They were incubated in LC3B primary antibody (Abcam, USA ab192890; the dilution ratio was 1:900) and dissolved in PBS with 1% BSA (Beyotime, China, ST023) and 0.3% Triton X-100 overnight at 4°C. The cells were washed with PBS 3 times (5 minutes each) and incubated in secondary antibody (ZSGB-BIO, China, ZF0511) for 2 hours at room temperature. After three additional washes in PBS, antifading mounting medium containing DAPI was applied (Solarbio, China, S2110). Images were acquired with a fluorescent inverted microscope (Nikon, Japan).

### Plasmid transfection

Cells were plated on 6-well plates 1 day before experimentation. LC3 plasmid (HedgehogBio, China, HH-LC3-040) was mixed with Lipo8000 Transfection Reagent (Beyotime, China, C0533) according to the manufacturer’s manual. After 48 hours, images were acquired with a fluorescent inverted microscope (Nikon, Japan).

### Xenograft mouse model

Female BALB/c nude mice (4-6 weeks old) were purchased from the Animal Ethics Committee of Chongqing Medical University and housed under specific pathogen-free (SPF) conditions with a 14-hour light/ 10-hour dark cycle. Two mice were subcutaneously injected with 1 × 10^7^ cells in the right thighs. After 4 weeks, the mice were sacrificed, and the xenografts were excised. Then, the xenografts were divided into equivalent pieces (approximately 32 mm^3^) and transplanted into the mesenteric artery at the cecum in another cohort of anesthetized mice. Four weeks later, the mice were sacrificed, and the xenografts were removed from the abdomens. The tumors were weighted and the tumor growth inhibition rate was calculated. All animal studies were approved by the Institutional Animal Care and Use Committee of the Chongqing Medical University.

### Statistical analysis

Except where otherwise noted, experiments were repeated at least 3 times. Statistical analysis was performed with Graph Pad Prism software using the Student *t* tests (for 2 groups). Differences between the values were considered statistically significant if *P* < 0.05. The results are presented as means ± SD.

## Supplementary Material

Supplementary Figures

Supplementary Table 1

Supplementary File 1
