# Supplementary material for: PRDX2 removal inhibits the cell cycle and autophagy in colorectal cancer cells
Source: Aging (Albany NY). 2020 Jul 20;12(16):16390–409. doi: 10.18632/aging.103690 (PMC7485722; doi:10.18632/aging.103690)
Supplement: Supplementary Figures [file aging-12-103690-s003..pdf]

SUPPLEMENTARY FIGURES

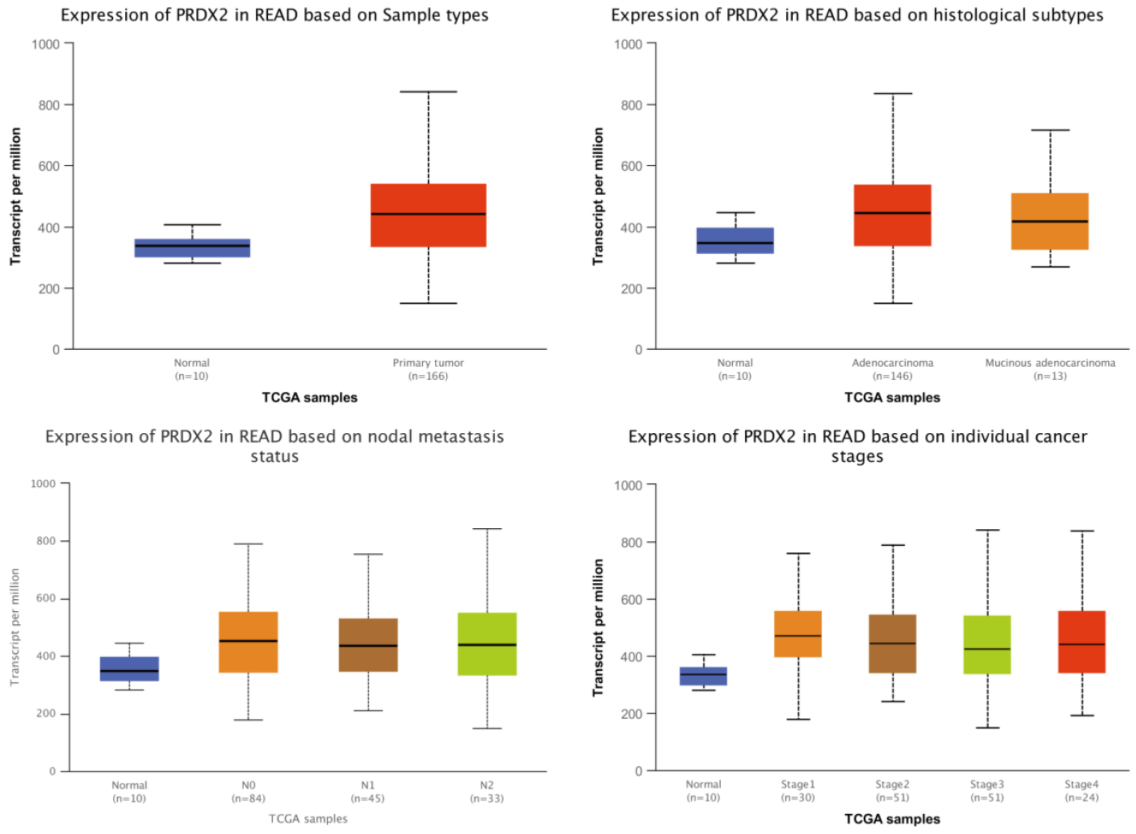

Supplementary Figure 1. Analysis of PRDX2 mRNA expression levels between READ and colon tissue according to histological subtypes, metastasis status, and individual cancer stages using the TCGA database through the UALCAN website.

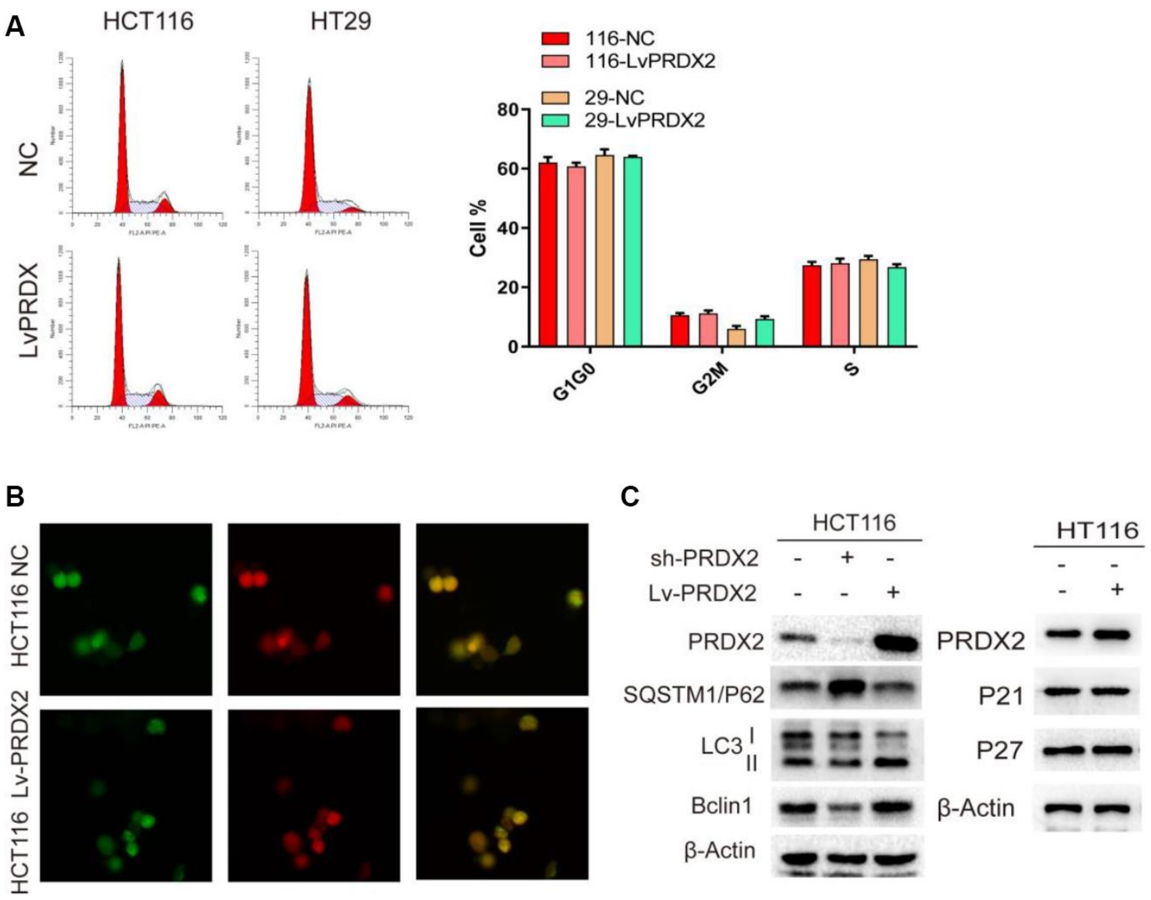

Supplementary Figure 2. Effects of PRDX2 over-expression on the cell-cycle and autophagy. (**A**) Flow cytometry cell-cycle analysis showed that lv-PRDX2 did not significantly affect the cell cycle. (**B**) NC and Lv-PRDX2 cells were infected with GFP-RFP-LC3 plasmids. (**C**) Western blots of proteins related to the autophagy and cell-cycle regulation in lv-PRDX2 and control CRC cells.

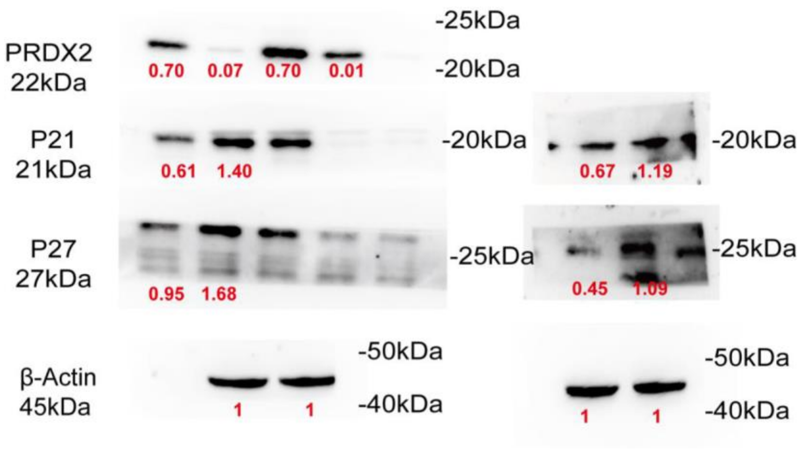

Supplementary Figure 3. Full-size blots of Figure 3C

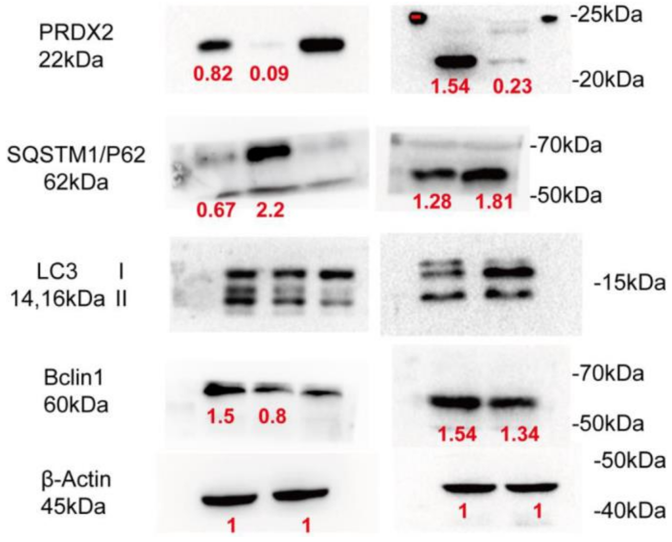

Supplementary Figure 4. Full-size blots of Figure 4D.

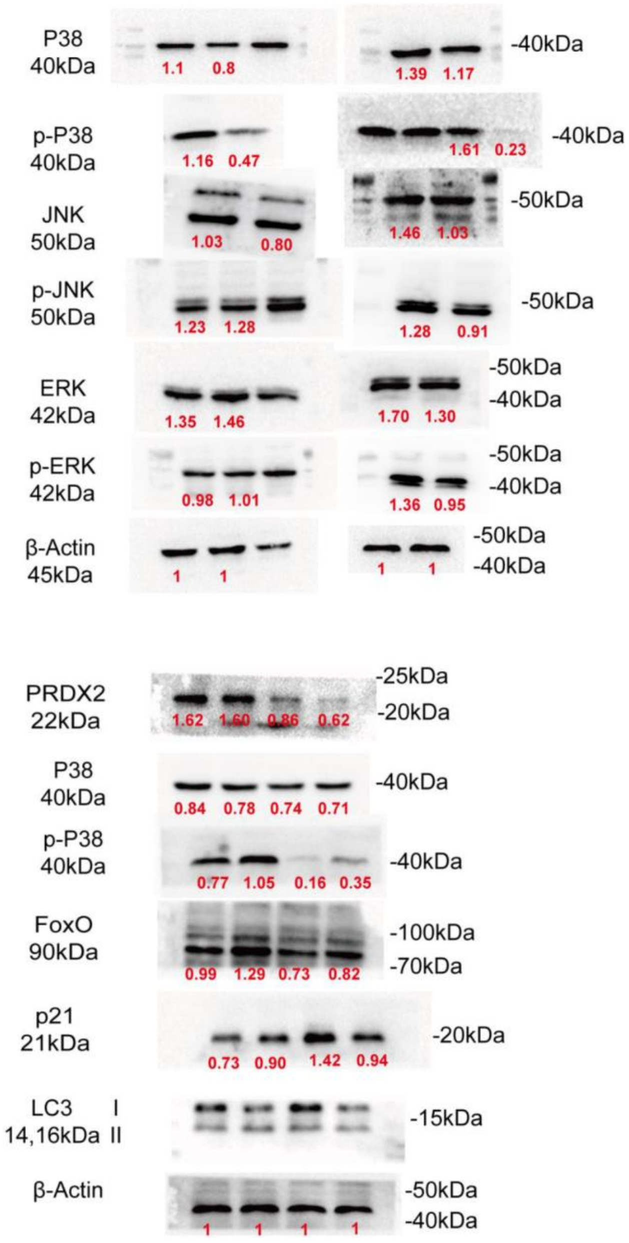

Supplementary Figure 5. Full-size blots of Figure 5A.

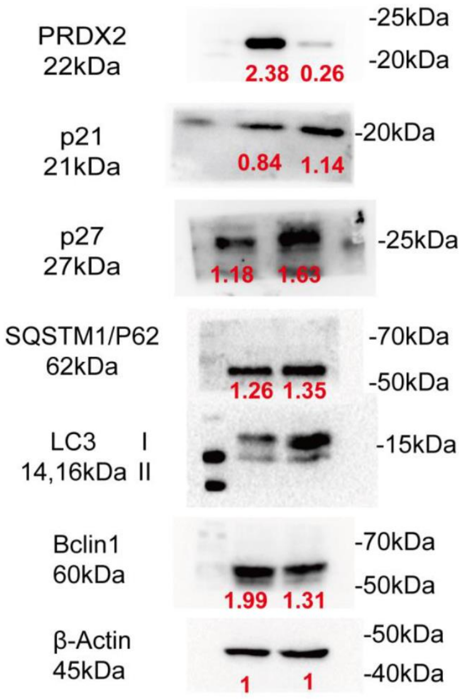

Supplementary Figure 6. Full-size blots of Figure5B.
