# Supplementary material for: PRDX2 removal inhibits the cell cycle and autophagy in colorectal cancer cells
Source: Aging (Albany NY). 2020 Jul 20;12(16):16390–409. doi: 10.18632/aging.103690 (PMC7485722; doi:10.18632/aging.103690)
Supplement: Supplementary Table 1 [file aging-12-103690-s001..pdf]

SUPPLEMENTARY TABLE

Supplementary Table 1. Antibodies for Western blotting.

Antibody	Company	Catalog	Dilution ratio
PRDX2	Proteintech	10545-2-AP	1:1000
P21	Cell Signaling Technology	2947	1:1000
P27	Bimake	A5053	1:1000
LC3	Abcam	ab192890	1:1000
P6/2SQSTM1	Proteintech	18420-1-AP	1:1000
Beclin1	Proteintech	11306-1-AP	1:1000
FOXO3A	Cell Signaling Technology	12829	1:1000
P38 MAPK	Cell Signaling Technology	8690	1:2000
phospho-P38 MAPK	Cell Signaling Technology	4511	1:1000
JNK	Cell Signaling Technology	9252	1:1000
phospho-JNK	Cell Signaling Technology	9255	1:1000
ERK	Cell Signaling Technology	4695	1:1000
phospho-ERK	Cell Signaling Technology	4370	1:1000
HRP-conjugated second antibody	Proteintech	SA00001-2	1:10000
